# The shortest distance between the skin and the peritoneal cavity is obtained with fascial elevation: a preliminary prospective laparoscopic entry study

**DOI:** 10.52054/FVVO.14.2.028

**Published:** 2022-07-01

**Authors:** G Kiran, I Yilmaz, S Aydin, F Sanlikan, E Ozkaya

**Affiliations:** Department of Ob/Gyn, Bezmialem Vakif University Faculty of Medicine, Istanbul, Turkey; Department of Ob/Gyn, Umraniye Training and Research Hospital, Istanbul, Turkey; Department of Ob/Gyn, Memorial Sisli Hastanesi, Istanbul, Turkey; Department of Ob/Gyn, Zeynep Kamil Women and Children’s Training and Research Hospital, Istanbul, Turkey.

**Keywords:** Laparoscopy, abdominal entry, fascial elevation, skin-to-fascia distance

## Abstract

The purpose of this study was to prospectively compare the measurement of skin-to-fascia distances in the neutral state, during manual elevation and by fascial elevation in patients who underwent laparoscopic surgery. In 53 patients, the distance between the skin and anterior wall of the rectus sheath was measured prospectively in following three different ways: (1) in neutral position, (2) during manual elevation and (3) during elevation of the fascia using forceps following an infraumbilical vertical skin incision. In all patients, subcutaneous tissue up to the fascia was dissected after a vertical skin incision. The skin-to-fascia distance of 30.9 mm (14.0-52.0 mm) in the neutral position decreased to 11.1 mm (0.0-26.0 mm) during the fascial elevation, while the mean distance increased to 40.1 mm (19-70 mm) during manual elevation (p < 0.001). In the closed laparoscopic entry technique in which a Veress needle is inserted into the peritoneum through a small incision, the needle should be introduced from the shortest distance between the skin and the peritoneum. Lifting the fascia with a proper surgical instrument in suitable patients could enable us to achieve this goal.

## Introduction

In minimally invasive surgery, access to the abdomen is the most important step prior to the intended surgery. More than half of the complications associated with the gastrointestinal system and main vascular structures in laparoscopic surgery occur during entry. In other words, a significant number of complications occur within the first few minutes of surgery (Semm and Semm, 1997; Lam et al., 2010; Harkki-Siren, 1999).

As is known, there are open and closed entry techniques (Vilos et al., 2017; Bemelman et al., 2000; Conliandolo et al., 1998). The umbilicus is the most preferred access point for closed entry in gynaecological minimally invasive surgery because the distance from the skin to the peritoneal cavity is the shortest at this site. In addition, the parietal peritoneum is adherent to the back of the fascia at this point; hence, the main factor determining the distance is the distance from the skin to the fascia. In this technique, after the skin incision, a Veress needle is inserted through the layers of the abdominal wall and then some safety tests are performed before gas insufflation. Although elevation of the abdominal wall by hand or forceps is often preferred prior to the Veress entry, direct lifting of the fascia is often neglected (Semm and Semm, 1997; Richardson and Sutton, 1999). Lifting the fascia was described in 1993 by Vakili and Knight, which is important because it makes it possible to lift the fascia higher and to keep it immobile (Vakili and Knight, 1993). The skin-to-fascia distance may vary greatly from patient to patient. While this distance is not too long in thin patients, it could be greater in patients with a high body mass index (BMI) due to an excess of subcutaneous fat tissue (Ulusoy et al., 2018). The distance to the fascia can be made less variable with a relatively simple technique during laparoscopic entry.

Although the fascia lifting technique has been described in the literature, studies showing how much the technique reduces the distance to the fascia quantitatively are very limited. The aim of this study was to determine the skin to fascia distance in neutral, manual elevation and fascial elevation objectively, as a shorter distance may facilitate entry and reduce failure.

## Materials and Methods

**Figure 1:**
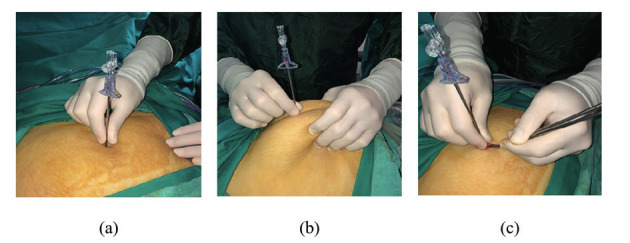
Measurement of skin-to-the anterior wall of rectus sheath distance using the tip of the Veress needle in neutral position (a); in manuel elevation (b) and during fascial elevation (c).

This study was conducted at a single gynaecology and obstetrics clinic of a university affiliated hospital in Istanbul, Turkey between 1 December 2017 and 31 August 2018. Institutional ethics committee approval was obtained for this study. Patient consent was not obtained because there was no additional procedure performed other than the non-invasive measurement of the skin-to- fascia distance during abdominal entry. Gravida, parity, abortion, BMI, vaginal birth, and number of caesarean deliveries in a group of patients planned for gynaecologic laparoscopy were recorded prospectively. Patients who had an umbilical hernia, previous midline incision, mass under the entry point or underwent umbilical hernia repair were excluded. The patients were placed in the lithotomy position under general anaesthesia, then a Foley catheter was inserted into the bladder and an uterine manipulator was placed into the uterus when indicated. Subcutaneous fat tissue was dissected up to the anterior rectus sheath after an infraumbilical vertical skin incision. The distance between the skin and the anterior wall of the rectus sheath was measured in the following three different ways: (1) in neutral position, (2) during manual elevation and (3) during fascial elevation with forceps. The measurement was made with a disposable ruler after the distance was determined by the tip of the Veress needle (Figure 1).

**Figure 2:**
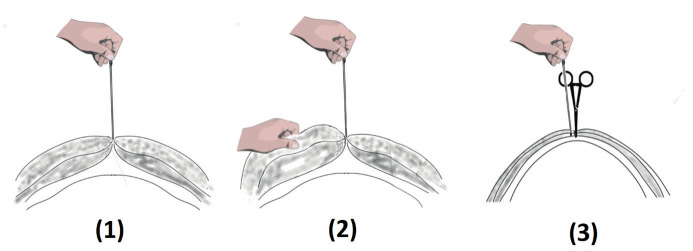
Illustration of the measurement methods used in this study: (1) in neutral position, (2)during manual elevation and(3) during fascia elevation by a forceps.

The observed rectus sheath was grasped with Pean forceps. While the rectus sheath was being lifted, the Veress needle was inserted perpendicularly at the tip of the forceps and then advanced into the peritoneal cavity. The illustration represents the measurement technique with the Veress needle in neutral position, during manual elevation and during fascial elevation with forceps (Figure 2).

After the aspiration and pressure test, insufflation was started. When the intraabdominal pressure reached 25 mmHg, the Veress needle was removed, and a 10 mm trocar was inserted into the abdomen. A camera was then placed, and ancillary trocars were introduced under direct vision. The pressure was reduced to 14 mmHg, and the patient was repositioned to the Trendelenburg position. The operation was continued at this pressure and position. The operation performed, the duration of operation time, and any entry complications were recorded.

Statistical analysis was performed with SPSS 17.0 software (SPSS Inc. Chicago, IL, USA). Normally distributed data were presented as mean with SD, and categorical outcomes were summarized using frequency distributions. The Wilcoxon signed rank test was used for quantitative variables. A p value of 0.05 was used as the cut-off point for significance.

## Results

A total of 66 patients planned for laparoscopic surgery were assessed for eligibility. A total of 13 patients were excluded from the study: eight patients with umbilical hernia, two patients with a history of previous midline incision, one patient with a large uterus, one patient with a subcutaneous umbilical lesion and one patient with a history of previous abdominoplasty. Alternative access sites were preferred in those patients. Finally, the data of 53 patients were evaluated. Demographic characteristics and operation details of the patients are given in Table I.

**Table I:** Patient demographics and procedure details.

Variable		Value
Number of patients		53
Age yr. mean (range)		47.2 (21.0 - 75.0)
BMI kg/m2, mean (range)		32.8 (19.1 - 69.9)
Gravida, mean (range)		3.7 (0.0 - 11.0)
Parity, mean (range)		2.7 (0.0 - 7.0)
Abortion, mean (range)		0.9 (0.0 - 4.0)
Surgery performed (n)		
	L/S HT ± BS/ BSO/ USO	37
	L/S cystectomy	9
	L/S BS/ BSO/ USO	2
	L/S HT + BSO + PPALND	2
	LT convertion HT + BSO	2
	L/S myomectomy	1
Entry time (skin- first trocar entry) min, mean (range)		4.9 (2.0 - 11.0)
Operation time (skin to skin) min, mean (range)		118.1 (45.0 - 476.0)

(HT: Hysterectomy; BS: Bilateral salpingectomy; BSO: Bilateral salpingo-oophorectomy; USO: Unilateral salpingo- oophorectomy; PPALND: Pelvic- para aortic lymph node dissection; LT: Laparotomy)

The mean time from the skin incision to the placement of the first trocar was 4.9 min (2.0-11.0 min). The mean duration of the operations was 118.1 min (45.0-476.0 min). No entry complication was observed, except for preperitoneal insufflation in one patient. The skin-to-fascia distance of 30.9 mm in the neutral position decreased to 11.1 mm in the fascial elevation, and this difference was statistically significant (p < 0.001). The mean distance during manual elevation increased to 40.1 mm. The distance between the fascial and manual elevation was also statistically significant (p < 0.001). In the subgroup analysis, patients were divided into two groups based on BMI: patients with a BMI ≥ 30 kg/m2 (n = 32) or those with a BMI < 30 kg/m2 (n = 21). The skin-to-fascia distances were 31.9 mm and 29.6 mm shorter in the fascial elevation group compared to the manual elevation group in the higher and lower BMI groups, respectively (Table II). The difference was statistically significant (p < 0.001).

**Table II:** Distance between skin to fascia and relation to body mass index.

	NP	ME	FE	
Mean distance (skin to facia) (mm)	30.9	40.1	11.1	<0.001
• BMI<30 (n=21)	25.2	37.0	7.4	<0.001
• BMI>30 (n=32)	34.7	44.7	12.8	<0.001

(NP: neutral position; ME: manuel elevation; FE: fascial elevation).

## Discussion

Our study demonstrated that by using the fascia elevation technique during laparoscopic access, the mean distance between skin and the fascia is 3 cm and 2 cm shorter than in manual elevation and in neutral position, respectively. Via reducing the distance that is needed for the Veress needle to pass, a more controlled and stable entry can be performed.

The technique of lifting the fascia at laparoscopic entry was first described in 1993 (Vakili and Knight, 1993). Insertion of the needle in the closed entry, providing the pneumoperitoneum, and then performing the primary trocar entry is the only procedure performed blindly in laparoscopy. This stage may be accompanied by some technical difficulties and complications. Repetitive access attempts may be required, especially in obese patients, due to decreased tactile sensation and uncertainty in the thickness of the preperitoneal layers. In cases where the panniculus is loosely attached to the anterior abdominal fascia, the preperitoneum, peritoneum, and even abdominal fascia may move deeply with the needle, making it difficult to reach the peritoneum. Repetitive attempts can be associated with some problems, including abdominal wall, preperitoneal, mesenteric, or omental emphysema (Vakili and Knight, 1993).

In the fascial fixation technique, after the skin incision, the anterior abdominal fascia is fixed using various clamps. During the passage of the needle, the instruments holding the fascia are pulled upwards, almost to the same level with the skin, so that the needle is applied with direct vision and excellent manual control. Although they are not completely safe during entry, various safety tests are performed to try to understand whether the needle is in the right place (Roy et al., 2001; Toeh et al., 2005). After adequate pneumoperitoneum is achieved, the primary trocar is inserted. A similar technique was previously defined by Mitchell et al. (Mitchell et al., 1991).

In our study, we aimed to measure how much advantage the technique provides in the distance of reaching the fascia compared to manual elevation and neutral position. Fascial elevation significantly shortened the distance compared to other techniques. In a study of 40 patients, Usta and colleagues compared the pneumoperitoneum distance obtained with clamps only with the depth obtained by additional manual elevation. They showed that an adequate pneumoperitoneum depth could be obtained with both techniques (3.9 mm and 4.5 mm, respectively) (Usta et al., 2017).

In an ultrasound study, it was shown that the average subcutaneous adipose tissue thickness was 3.1 cm in a group of patients. In our study, if the fascia was lifted by a surgical instrument, an approximately 3 cm shorter distance was traversed in comparison to the manual elevation group; almost equal to the thickness of the adipose tissue (De Lucia Rolfe et al., 2010).

In their study, Habibi et al. showed that direct trocar entry with elevation of the rectus sheath was safe and effective in a group of obese patients planned for bariatric surgery (Habibi et al., 2017).

Similarly, our results showed that the effect of rectus sheath elevation causing entry distance shortening in patients with a low BMI persists in patients with a high BMI.

Intraumbilical entry may not be appropriate for patients with an umbilical hernia. In those patients, visceral organs may be damaged during fascial detection. Otherwise, this technique can be used safely in all patients without any further contraindications to umbilical entry.

In order to compare the superiority of any technique for laparoscopic entry, it is necessary to work with a very large patient group. The major limitation of the study is due to the small number of patients. Therefore, our report may be considered a preliminary investigation. The strengths of our study are that all operations were performed by the same surgeon (G. K.) and the study was performed prospectively in a single centre.

Access to the abdominal cavity is the mandatory step of minimally invasive surgery, regardless of whether laparoscopic or robotic surgery is performed. In the present study, the patients in whom intraumbilical entry was preferred were evaluated. We use fascial fixation with an appropriate surgical instrument during abdominal entry in patients in which other entry points (such as Lee-Huang or Palmer’s point) are preferred for various reasons (Lee et al., 2001; Agarwala and Liu, 2005).

Although no comparison was made with these patients, we have observed in many patients that lifting the fascia can be used successfully at these alternative entry points. It should be noted that shortening the distance between the skin and the fascia does not guarantee safe entry. The advantages of fascia lifting technique should be supported by further and larger studies to improve access safety.

## References

[1] Agarwala N, Liu CY (2005). Safe entry techniques during laparoscopy: left upper quadrant entry using the ninth intercostal space--a review of 918 procedures.. J Minim Invasive Gynecol.

[2] Bemelman WA, Dunker MS, Busch OR (2000). Efficacy of establishment of pneumoperitoneum with the Veress needle, Hasson trocar, and modified blunt trocar (TrocDoc): a randomized study.. J Laparoendosc Adv Surg Tech A.

[3] Cogliandolo A, Manganaro T, Saitta FP (1998). Blind versus open approach to laparoscopic cholecystectomy: a randomized study.. Surg Laparosc Endosc.

[4] De Lucia Rolfe E, Sleigh A, Finucane FM (2010). Ultrasound measurements of visceral and subcutaneous abdominal thickness to predict abdominal adiposity among older men and women.. Obesity (Silver Spring).

[5] Habibi M, Seyit H, Kones O (2017). Direct Trocar Insertion with Elevation of the Rectus Sheath in Bariatric Surgery: A Novel Technique.. PolPrzegl Chir.

[6] Harkki-Siren P (1999). The incidence of entry-related laparoscopic injuries in Finland.. Gynecol Endosc.

[7] Lam A, Khong SY, Bignardi T (2010). Principles and strategies for dealing with complications in laparoscopy.. Curr Opin Obstet Gynecol.

[8] Lee CL, Huang KG, Jain S (2001). A new portal for gynecologic laparoscopy.. J Am Assoc Gynecol Laparosc.

[9] Mitchell MB, Stiegmann GV, Mansour A (1991). Improved technique for establishing pneumoperitoneum for laparoscopy. Surg Laparosc Endosc.

[10] Richardson RE, Sutton CJG (1999). Complications of first entry: a prospective laparoscopy audit.. Gynaecol Endosc.

[11] Roy GM, Bazzurini L, Solima E (2001). Safe technique for laparoscopic entry into the abdominal cavity.. J Am Assoc Gynecol Laparosc.

[12] Semm K, Semm I (1997). Safe insertion of trocars and the Veress needle using standard equipment and the 11 security steps.. Gynec Endosc.

[13] Teoh B, Sen R, Abbott J (2005). An evaluation of four tests used to ascertain Veres needle placement at closed laparoscopy.. J Minim Invasive Gynecol.

[14] Ulusoy S, Özer M, Kılınç I, Parlak Ö (2018). Direct trocar entry for laparoscopy safety and efficiency.. Laparosc Endosc Surg Sci.

[15] Usta TA, Karacan T, Kovalak EE (2017). Is there any difference between the distances created by towel clamp lifting and towel clamp plus manual lifting of the anterior abdominal Wall for direct trocar entry in laparoscopic gynecologic surgery? A prospective interventional study.. J Turk Ger Gynecol Assoc.

[16] Vakili C, Knight R (1993). A technique for needle insufflation in obese patients. Surg Laparosc Endosc.

[17] Vilos GA, Ternamian A, Dempster J (2017). No. 193- Laparoscopic entry: A review of techniques, technologies, and complications. J Obstet Gynaecol Can.

